# Prescribed computer games in addition to occlusion versus standard occlusion treatment for childhood amblyopia: a pilot randomised controlled trial

**DOI:** 10.1186/s40814-015-0018-y

**Published:** 2015-06-11

**Authors:** Vijay K. Tailor, Selina Glaze, Payal Khandelwal, Alison Davis, Gillian G. W. Adams, Wen Xing, Catey Bunce, Annegret Dahlmann-Noor

**Affiliations:** 1 NIHR Biomedical Research Centre at Moorfields Eye Hospital and UCL Institute of Ophthalmology, 162 City Road, London, EC1V 2PD UK; 2 Moorfields at Bedford Hospital NHS Trust, Kempston Road, Bedford, MK42 9DJ UK; 3 South Essex Partnership Foundation Trust, Enhanced Services Centre, 3 Kimbolton Road, Bedford, MK40 2NT UK

**Keywords:** Amblyopia, Child, Clinical trial

## Abstract

**Background:**

Amblyopia (“lazy eye”) is the commonest vision deficit in children. If not fully corrected by glasses, amblyopia is treated by patching or blurring the better-seeing eye. Compliance with patching is often poor. Computer-based activities are increasingly topical, both as an adjunct to standard treatment and as a platform for novel treatments. Acceptability by families has not been explored, and feasibility of a randomised controlled trial (RCT) using computer games in terms of recruitment and treatment acceptability is uncertain.

**Methods:**

We carried out a pilot RCT to test whether computer-based activities are acceptable and accessible to families and to test trial methods such as recruitment and retention rates, randomisation, trial-specific data collection tools and analysis. The trial had three arms: standard near activity advice, Eye Five, a package developed for children with amblyopia, and an off-the-shelf handheld games console with pre-installed games. We enrolled 60 children age 3–8 years with moderate or severe amblyopia after completion of optical treatment.

**Results:**

This trial was registered as UKCRN-ID 11074. Pre-screening of 3600 medical notes identified 189 potentially eligible children, of whom 60 remained eligible after optical treatment, and were enrolled between April 2012 and March 2013. One participant was randomised twice and withdrawn from the study. Of the 58 remaining, 37 were boys. The mean (SD) age was 4.6 (1.7) years. Thirty-seven had moderate and 21 severe amblyopia. Three participants were withdrawn at week 6, and in total, four were lost to follow-up at week 12. Most children and parents/carers found the study procedures, i.e. occlusion treatment, usage of the allocated near activity and completion of a study diary, easy. The prescribed cumulative dose of near activity was 84 h at 12 weeks. Reported near activity usage numbers were close to prescribed numbers in moderate amblyopes (94 % of prescribed) but markedly less in severe amblyopes (64 %). Reported occlusion usage at 12 weeks was 90 % of prescribed dose for moderate and 33 % for severe amblyopes.

**Conclusions:**

Computer-based games and activities appear acceptable to families as part of their child’s amblyopia treatment. Trial methods were appropriate and accepted by families.

## Background

With a prevalence of between 2 and 5 %, amblyopia is the commonest visual deficit in children in the UK [1, 2]. In developing countries, amblyopia is the second most common cause of functional low vision in children [3]. It is a developmental defect; the most common risk factors are anisometropia (a difference in glasses prescription between the two eyes) and strabismus (misalignment of the visual axes). The imbalance of input to the brain from the two eyes results in a range of vision defects such as a reduction in acuity (resolution) and abnormal binocular function (poor stereopsis/3D vision). Unless treated early, amblyopia is irreversible. Accidents affecting the better-seeing eye can lead to a loss of quality of life and independence [4].

Based on previous randomised controlled trials (RCT) amblyopia is treated in a stepwise approach. Visually significant refractive errors are corrected by wearing glasses; this initial period of “refractive adaptation” or “optical treatment” may extend to 16–18 weeks [5–7]. If residual amblyopia persists, the better-seeing eye is patched for 2 or 6 h a day, depending on severity [8, 9]. Blurring of vision by atropine drops may be as effective [10]. Many units in the UK offer parents the choice of patching or atropine, but many parents favour occlusion, because treatment duration is more precise (i.e. only when the patch is on) and because of safety concerns over permanently blurring the vision in the better-seeing eye [11, 12].

Even with best treatment, not all children achieve normal visual acuity in the amblyopic eye. Only around 25 % of those with severe amblyopia and 58 % of those with moderate amblyopia improve to a level of 6/9 (0.18 logMAR) or better [13, 14], reflecting the need for refinement of existing and development of new treatments. Compliance is a significant barrier. Children attempt to remove the patch, and parents may feel guilty about exposing their child to occlusion. Compliance ranges from 41–57 % [15–17]. Compliance may be less with greater prescribed daily dose and greater severity of amblyopia [15], prolonged treatment duration and lower appointment attendance [17]. Better education of families may increase compliance [16]. A broader approach based on current knowledge [18] as well as further research to offer individualised treatments could further improve amblyopia treatment.

Near activities such as drawing, colouring, or playing computer games during patching are generally considered to improve treatment effectiveness and were included in a previous RCT [19]. A recent RCT investigating the role of near activities did not demonstrate an effect on visual outcome but did not standardise near activities [20].

Computer games may be an attractive near activity to enhance occlusion treatment: parents can choose to allow games only during occlusion, games distract the child, and they provide the child with a highly repetitive fine visual task. However, some parents are concerned about allowing their children daily access to computers and computer games, potentially limiting the use of this technology in amblyopia treatment. It is increasingly important to address this question, as the last few years have seen the development of novel amblyopia treatment approaches using dichoptic image presentation on personal or tablet computers or mobile phones as a platform for games or movies [21–25]. Whilst these technologies have not yet been tested in an RCT, the lay press has reported that simple off-the-shelf games may improve visual acuity by encouraging wearing of the occlusion patch and improving compliance [26]. However, standard games use visual stimuli difficult to discern for amblyopic eyes. Moorfields Eye Hospital has developed a package of educational and computer- and paper-based near activities specifically for children with amblyopia, centred on a team of cartoon space cadets, the “Moorfields’ Eye Five” (www.eyesite.nhs.uk). The online games can be accessed from any home or tablet computer, and targets are of bigger size than in standard games, so they are easier to detect by amblyopic eyes. Storylines for parents/carers to read to children are designed to engage children and to make them interested in the characters they can colour in. A paper-based sticker chart is used as a record of daily successful patching.

We aimed to determine whether computer-based near activities, either off-the-shelf or custom-designed for amblyopia, improve adherence to occlusion treatment. However, as many parameters surrounding study design and feasibility, particularly recruitment and treatment acceptability to families, were unknown, we carried out a pilot trial for a later phase III randomised controlled trial. The aims of this pilot trial are to test recruitment and retention rates, acceptability and accessibility of computer games to children and parents/carers, randomisation, trial-specific data collection tools and analysis.

## Methods

This RCT was approved by the National Research Ethics Committee London—London Bridge, and registered on the UKCRN portfolio database as UKCRN-ID 11074.

### Study design

We conducted an observer-masked, parallel-group RCT, randomising a total of 60 children age 3–8 years with unilateral amblyopia to either the Moorfields’ Eye Five Package or a game on a handheld Nintendo 3DS console or standard occlusion treatment, using a 1:1:1 allocation ratio. The sample size of 20 participants per arm is commonly used in pilot trials [27].

### Study setting

We identified children between April 2012 and March 2013 at clinics at Moorfields Eye Hospital, Moorfields at Bedford Hospital, the South Essex Partnership Foundation Trust Orthoptic Community Eye Clinics in Bedford and the Homerton Hospital. We pre-screened the medical notes of all newly referred children to identify those referred for reduced vision and/or strabismus.

### Inclusion criteria

We included children age 3–8 years with newly diagnosed anisometropic, strabismic or combined mechanism amblyopia. Children had completed a period of optical treatment, and a clinical indication to start additional treatment had been made. Parents/carers had been given a choice between occlusion or pharmacological blurring and had decided that they would prefer occlusion treatment. Children had an interocular difference in best corrected visual acuity (BCVA) at least 0.20 logMAR and no previous ophthalmic treatment other than glasses. All children had access to a desktop, laptop or tablet computer at home, with variable screen size.

### Recruitment

As part of their clinical management, all children underwent comprehensive orthoptic and ophthalmic assessment including cycloplegic refraction and fundoscopy; glasses were prescribed as appropriate. BCVA was monitored at intervals of 6–10 weeks. We defined the end of “optical treatment only” as BCVA not improving on two consecutive visits despite reportedly good compliance. If children were eligible at the end of optical treatment, we gave families verbal and written information about the trial. Following at least 2 weeks to consider participation, a research orthoptist with training in Good Clinical Practice explained the study procedures, addressed any questions and obtained written parental/carer consent at the subsequent clinic visit; children gave verbal assent; written assent was optional.

### Baseline assessment

The study orthoptist carried out a baseline assessment of BCVA and stereopsis on an age-appropriate test. BCVA was measured using Thompson V2000 software which displays HOTVX letters or Kay pictures at 3 m or handheld Keeler or Kays crowded logMAR charts. Visual acuity was recorded in logMAR. Stereopsis was measured using Frisby, TNO or the Titmus fly test and recorded in seconds of arc. Whilst tests varied between participants, each participant had the same test for visual acuity and stereoacuity at all timepoints.

### Randomisation/allocation

A randomisation schedule was prepared based on permuted blocks of varying sizes by a data manager within the R & D department. When a child had been recruited to the trial, the orthoptist telephoned the data manager to find out what the next treatment on the schedule was. It was not possible to mask families to the treatment, but research staff was masked where possible, e.g. data were collected by staff who did not know which treatment participants were receiving, and the participants were asked not to disclose their treatment to the examining health professionals.

### Interventions

As experimental interventions, we used (1) a Super Mario World game on a handheld Nintendo 3DS console and (2) the Moorfields’ Eye Five Package, a web-based programme of educational and near activities for children with amblyopia. The package combines a cartoon story book, paper-based activities such as colouring pages and puzzles, and internet-based computer games (http://www.eyesite.nhs.uk/). Children who were allocated the Super Mario game received a Nintendo console with the game; those in the Eye Five group were given a paper activity and sticker book and were asked to access the online games via a computer at home. The comparator was standard verbal and written instructions on near activities such as colouring, reading and writing.

The study orthoptist gave participants and parents/carers information about the use of the allocated near activity which was prescribed for 1 h whilst wearing the occlusion patch. Children were asked not to use any other consoles during patching. Children with moderate amblyopia (BCVA in the amblyopic eye better than 0.6 logMAR) were prescribed 2 h of occlusion per day; participants with severe amblyopia (BCVA in the amblyopic eye 0.6 logMAR or worse), 6 h.

### Post-randomisation assessments

We asked parents to complete a diary to monitor adherence and ease of use of near activities and patching, and reviewed the diaries at 6 and 12 weeks after randomisation. We asked children and parents/carers about any adverse events and monitored BCVA in the fellow eye to detect a reversal of amblyopia. At 12 weeks, we asked children and parents/carers four questions about the ease of occlusion, use of the near activity, use of parental diaries and the effect of occlusion on the participant’s self-esteem, using a five-point Likert scale. These questions aimed to explore acceptability of the interventions and the study design to children and families.

Orthoptists masked to the allocated treatment carried out assessments of BCVA and stereoacuity at 6 and 12 weeks. All data were collected on paper-based case report forms completed at each treatment visit and parental diaries completed daily.

### Outcome measures

Primary outcome measures related to study methodology and feasibility [28]: recruitment and retention rates and acceptability of the experimental interventions. Secondary outcomes included adherence to experimental and standard treatments, BCVA in the amblyopic eye, stereopsis and adverse events. These measures were to be used to estimate the treatment effect size for a sample size calculation for a subsequent phase III RCT.

### Sample size and statistical methods

The sample size of 20 per treatment arm is commonly used in pilot trials [27].

STATA version 12 was used to perform data analysis. Baseline characteristics were summarised by treatment group to assess the adequacy of the randomisation. Numbers and proportions were used for categorical variables, means and standard deviations (SD), or medians and interquartile ranges (IQR) were used for continuous variables depending on whether the data appeared to be normally distributed. For each participant, the number of hours of occlusion and of near activity use was calculated by adding up the figures recorded in the parent/carer diaries. For number of hours of occlusion and near activities (adherence measures) and for BCVA and stereopsis (visual outcomes), we calculated the median and IQR.

## Results

### Primary outcomes: recruitment and retention rates and acceptability of study methodology and interventions

#### Recruitment

**Fig. 1:**
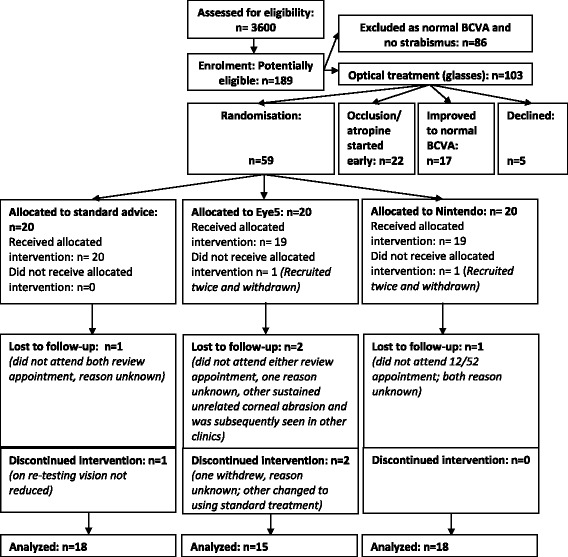
Consort flowchart of recruitment, allocation of intervention and 12-week analysis

We pre-screened the medical notes of 3600 children, identifying 189 potentially eligible patients referred for reduced vision and/or strabismus (Fig. 1). Following screening and discussion of the study with eligible families, we enrolled 60 children over a period of 12 months (April 2012–March 2013). All participants were recruited on the day when the decision to start occlusion treatment was made.

One patient was enrolled twice, by different study orthoptists and was allocated to different treatment arms. A review of enrolment, randomization and clearer identification of trial patients prevented further double-enrolments. The participant was excluded from follow-up, resulting in a loss of two recruitment numbers, as the family may have chosen not to reveal the first allocation in order to receive a different treatment.

The period of optical treatment caused a slow start to recruitment into the trial; in the first 5 months of the trial, an average of 1.2 (SD 1.1) children were recruited per month; in the remaining trial period, this rose to a mean of 7 (SD 2.6) per month.

#### Demographical and clinical characteristics of study population

Of the 58 remaining participants, 37 were boys and 21 girls (Table 1). The mean (SD) age was 4.6 (1.7) years. Thirty-seven had moderate and 21 severe amblyopia. Median (IQR) BCVA in the amblyopic eye at baseline was 0.5 (0.34–0.7) logMAR. 24 (41 %) children had anisometropic, 16 (28 %) strabismic and 16 (28 %) combined mechanism amblyopia. In 2 (3 %) cases the diagnosis was not clear.

**Table 1:** Participant characteristics

	Standard (*n* = 20)	Eye five (*n* = 19)	Nintendo (*n* = 19)
Gender, *n* (col %)	13(65)	12(63)	12(63)
Males	7(35)	7(37)	7(37)
Females			
Age (years), median (IQR)	5.1(3.5–6)	5.1(3.5–6.1)	4.3(4–5.4)
Type of amblyopia *n* (col %)	5(25)	11(57.9)	8(42.1)
Anisometropic	6(30)	5(26.3)	5(26.3)
Strabismus	8(40)	2(10.5)	6(31.6)
Mixed	1(5)	1(5.3)	
Unknown			
Severity of Amblyopia	11(55)	13(68.4)	13(72.2)
Moderate	9(45)	6(11.6)	6(27.8)
Severe			

#### Randomisation and allocation of interventions

Central randomisation was effective; there were no delays in allocating interventions.

#### Retention/dropout

One child was enrolled and randomised twice, to different arms, and subsequently withdrawn from the study (Fig. 1). Four participants were lost to follow-up at week 12: three did not attend without giving reasons, and one sustained an unrelated corneal abrasion and was transferred to a different clinic. Three participants were withdrawn at W6: one because their unaided vision was normal on retesting and no amblyogenic risk factors were present, one withdrew without giving reasons, and one discontinued the intervention (Eye Five) and started using standard treatment, as the family felt computer games to be too intrusive to family life. Minor protocol deviations concerned children using the Eye Five website on tablet computers rather than desktop PCs. This may influence the apparent size of objects but may be compensated for by a reduced viewing distance if tablets are held closer to the eye.

#### Accessibility and acceptability of interventions and study procedures

Fifty-one of 58 families (88 %) completed the diaries. In all groups, most children and parents/carers found completion of the diary and usage of the allocated near activity moderately to very easy. Those diaries which were completed were completed in full. No comments or adverse events were noted. Families also reported occlusion treatment from moderately to very easy. The effect of patching on the child’s self-esteem was reported as between slight and moderate. The results were similar between treatment groups.

### Secondary outcomes

#### Adherence to prescribed near activity

The prescribed cumulative dose of near activity was 42 h at 6 weeks and 84 at 12. Reported usage numbers were close to prescribed numbers in moderate amblyopes (94 % of prescribed) but markedly less in severe amblyopes (64 %). Figures were similar across treatment groups. Table 2 summarises compliance data.

**Table 2:** Hours spent on prescribed near activity and reported hours of occlusion treatment at the 6- and 12-week review

Compliance median (IQR)	Standard (*N* = 20)	Eye 5 (*N* = 19)	Nintendo (*N* = 19)	Total (*N* = 58)
Cumulative hours of near activity				
Moderate amblyopia 0–6 wks	42 (21–48), *n* = 11	39 (11–43), *n* = 12	42 (32–54), *n* = 12	42 (21–48), *n* = 35
Severe amblyopia 0–6 wks	23 (8–39), *n* = 8	29 (9–42), *n* = 5	12 (8–18), *n* = 5	20 (8–40), *n* = 18
Moderate amblyopia 0–12 wks	77 (63–84), *n* = 10	76 (30–84), *n* = 10	79 (68–99), *n* = 12	79 (61–84), *n* = 32
Severe amblyopia 0–12 wks	49 (24–81), *n* = 8	70 (34–82), *n* = 4	24 (17–118), *n* = 5	54 (17–82), *n* = 17
Cumulative hours of occlusion				
Moderate amblyopia 0–6 wks	84 (60–88), *n* = 11	66 (36–82), *n* = 12	74 (46–82), *n* = 12	72 (51–84), *n* = 35
Severe amblyopia 0–6 wks	74 (64–145), *n* = 8	84 (61–94), *n* = 5	96 (86–126), *n* = 5	85 (64–98), *n* = 18
Moderate amblyopia 0–12 wks	158 (126–180), *n* = 10	139 (97–164), *n* = 10	156 (93–168), *n* = 12	153 (108–168), *n* = 32
Severe amblyopia 0–12 wks	190 (141–292), *n* = 8	146 (125–159), *n* = 4	351 (160–432), *n* = 5	168 (142–351), *n* = 17

*N* = total number of patients in the study arm, *n* = number of patients in the group with valid data (only shown when there is invalid or missing data); The prescribed numbers of hours of near activity use were 42 at 6 and 84 at 12 weeks. Children with moderate amblyopia used the prescribed near activity markedly more than children with severe amblyopia. The prescribed numbers of hours of occlusion at 6 weeks were 84 for moderate and 252 for severe amblyopia and at 12 weeks 168 and 504 h, respectively. Despite the difference in prescribed hours, effective hours reported in the diaries were similar in children with moderate and severe amblyopia

*IQR* interquartile range

#### Adherence to occlusion

The prescribed numbers of hours of occlusion at 6 weeks were 84 for moderate and 252 for severe amblyopia and at 12 weeks, 168 and 504 h, respectively. Despite the difference in prescribed hours, effective hours reported in the diaries were similar in children with moderate and severe amblyopia. At 12 weeks, children with moderate amblyopia had patched 90 % of the prescribed time, but those with severe amblyopia had only received 33 % of the prescribed dose.

#### Visual outcomes

Of 58 participants, data on BCVA were available in 53 at the 6- and 51 at the 12-week timepoint (91 and 88 %), and stereopsis data were available in 52 patients at 6 weeks and 50 at 12 weeks (90 and 86 %). At 12 weeks data were available for 18 in the standard group, 15 in the Eye Five group and 18 in the Nintendo Group. BCVA improved at both 6 and 12 weeks in all three groups. At 12 weeks, median BCVA in children with severe amblyopia in the standard advice group improved by 0.27, in the Eye Five group by 0.26 logMar and in the Nintendo group by 0.26 logMar. In children with moderate amblyopia, median BCVA improved by 0.08, 0.2 and 0.26 logMar, respectively. Table 3 summarises visual outcome data.

**Table 3:** BCVA and stereoacuity at 6 and 12 weeks

Visual outcomes	Standard (*n* = 20) Median (IQR)			Eye 5 (*n* = 19) Median (IQR)			Nintendo(*n* = 19) Median (IQR)		
Baseline BCV	Severe	Moderate	None	Severe	Moderate	None	Severe	Moderate	None
Amblyopic or non-amblyopic eyes	*n* = 9	*n* = 11	*n* = 20	*n* = 5	*n* = 13	*n* = 19	*n* = 6	*n* = 13	*n* = 18
	0.8 (0.8–1)	0.36 (0.32–0.5)	0.1 (0.01–0.12)	0.76 (0.7–0.78)	0.35 (0.3–0.5)	0.1 (0.05–0.14)	0.8 (0.7–0.9)	0.4 (0.32–0.46)	0.1 (0.02–0.12)
6 weeks VA	Severe	Moderate	None	Severe	Moderate	None	Severe	Moderate	None
Amblyopic or non-amblyopic eyes	*n* = 8	*n* = 11	*n* = 19	*n* = 5	*n* = 12	*n* = 17	*n* = 5	*n* = 12	*n* = 17
	0.6 (0.59–0.89)	0.22 (0.06–0.4)	0.1 (0–0.15)	0.62 (0.58–0.75)	0.25 (0.19–0.31)	0.06 (0.05–0.12)	0.5 (0.5–0.7)	0.25 (0.2–0.38)	0.1 (0–0.1)
12 weeks VA	Severe	Moderate	None	Severe	Moderate	None	Severe	Moderate	None
Amblyopic or non-amblyopic eyes	*n* = 8	*n* = 10	*n* = 18	*n* = 4	*n* = 11	*n* = 15	*n* = 6	*n* = 12	*n* = 18
	0.53 (0.47–0.73)	0.28 (0.12–0.4)	0.1 (0–0.14)	0.5 (0.46–0.59)	0.15 (0.1–0.2)	0.06 (0.02–0.1)	0.54 (0.5–0.58)	0.14 (0.1–0.31)	0.01 (0–0.1)
Stereopsis									
Baseline	0 (0–155)			110 (0–170)			110 (0–480)		
6 weeks	0 (0–170), *N* = 19			85 (0–170), *N* = 16			110 (70–170), *N* = 17		
12 weeks	0 (0–100), *N* = 18			85 (0–100), *N* = 14			85 (0–110), *N* = 18		

*N* = total number of patients in the study arm, *n* = number of patients in the group with valid data (only shown when there is invalid or missing data); At 12 weeks, median BCVA in children with severe amblyopia in the standard advice group improved by 0.27, in the Eye Five group by 0.26 logMar and in the Nintendo group by 0.26 logMar. In children with moderate amblyopia, median BCVA improved by 0.08, 0.2 and 0.26 logMar, respectively. Change in stereoacuity was small, if any

*IQR* interquartile range

#### Adverse events

The only reported adverse event was intrusiveness of computer games on family life in one participant.

## Discussion

This pilot trial addresses the question whether a full phase III trial of computer-based near activities would be feasible, i.e. whether families would accept computer-based games and activities as part of their child’s amblyopia treatment and whether the study methods were acceptable. The pilot demonstrated a number of methodological weaknesses, but improved methods, recruitment and acceptability data show that a future RCT would be feasible.

The challenges we encountered may inform future trials in this field. Recruitment was initially slow due to pre-enrolment optical treatment. In addition, 17 % of children in our cohort improved on optical treatment to a point where they no longer met the eligibility criteria for this trial. This figure is consistent with other series (10 to 27 %) [29, 6, 7]. Seven of 58 children (12 %) did not complete the study; these figures will inform sample size calculations for future trials. The interventions were acceptable to families; only one participant withdrew from the study due to computer games being disruptive to family life. This is greatly encouraging for the planning of future trials, such as those involving computer-based dichoptic image presentation.

Of note, some children accessed web-based games via tablet computers rather than desktops or laptops. The Eye Five activities were developed to encourage use by children with amblyopia by presenting larger targets than off-the-shelf games. This trial shows that children enjoyed playing these games, and a wider range of games and activities is now in preparation. As children can compensate for smaller object size on tablet computer screens by holding the screen closer to their eyes, future studies should allow use of any computer as part of the protocol.

Diaries to measure compliance were well accepted by families; 88 % were completed. Ideally, compliance should be measured objectively by occlusion dose monitors (ODM) [6, 30], as the effective occlusion dose may be less than 50 % of the prescribed dose [17]. When our trial was set up, ODMs were not commercially available, and developing ODMs exceeded the study budget. We therefore opted for parental/carer diaries. Pragmatic amblyopia treatment studies have reported outcomes based on prescribed occlusion dose [13, 14, 31]. The reported usage of near activities and occlusion overall was as expected from previous reports, confirming the validity of this approach.

This pilot study also provides preliminary data on compliance with treatment and visual outcomes. Compliance with occlusion as reported by diaries appeared similar between groups. Children with moderate amblyopia received 90 % of the prescribed patching dose but those with severe amblyopia only 33 %. This means that those with severe amblyopia effectively patched for the same amount of time per day as moderate amblyopes, i.e. 2 h. Our figures are more extreme than those reported by other studies which report 41–57 % [15–17]. The only other RCT that specifically explored the use of near activities prescribed 2 h of patching and near activity to children with moderate and severe amblyopia and observed that children received 95 % of the prescribed regime [20]. The children in our study effectively received the same dose. Interestingly, visual outcomes are also similar; as this RCT reported mean improvement of BCVA of 2.5 at 8 and 2.9 lines at 17 weeks [20]. Another secondary aim was to estimate treatment effect size on visual outcomes for future sample size calculations. The “gold standard” method to measure visual acuity in adults is the ETDRS letter chart, which is based on linear presentation and letter-by-letter logarithmic scoring. The equivalent “gold standard” for children is a simplified crowded logMAR test, such as the Keeler crowded logMAR chart (originally described as “Glasgow Acuity Cards” [32, 33]. This test has a 95 % confidence interval for test-retest variability of 0.1 logMAR [33], which we selected as minimum clinically important difference which we wished to detect. Younger children may not co-operate letter recognition on the Keeler crowded logMAR test; hence, the crowded Kay Pictures test is often used in children age 2 to 4 years, though data on test reliability in children are sparse [34–36]. When we started this study, we considered that any chart giving a logMAR visual acuity measurement would be acceptable for this pragmatic study. However, due to the lack of validation of tests for younger children, a definitive clinical trial would be more rigorous if the “gold standard” acuity test was carried out on all children and at all timepoints. This would mean that children who cannot co-operate with this test cannot enter the study.

The use of different test charts is a significant limitation of the present, pragmatic pilot trial and may impact the sample size calculation for future trials, but our findings with regards to improvements in visual acuity are similar to those reported by previous studies. Overall, BCVA improved by 0.26 to 0.27 logMAR in severe and 0.08 to 0.26 logMAR in moderate amblyopes. This is similar to previous figures of 0.12–0.35 logMAR [29, 6, 13, 14].

Considering the data on controls subjects in our study only, the mean change in BCVA was 0.21 (SD 0.13). For a study with two arms, a sample size of 32 in each group will have 85 % power to detect a difference in means of 0.100 (the difference between a Group 1 mean, μ1, of 0.210 and a Group 2 mean, μ2, of 0.110) assuming that the common standard deviation is 0.130 using a two group *t* test with a 0.050 two-sided significance level. Allowing for possible loss to follow-up of 5 %, a total sample size of 68 children would be required.

Since we conducted this pilot trial, binocular treatment approaches have become available for adults and children with amblyopia [21–25]. We therefore decided not to proceed to a full phase III trial of computer games to enhance occlusion but have used the feasibility data from this pilot to design a trial of a binocular treatment.

## Conclusions

Computer-based games and activities appear acceptable to families as part of their child’s amblyopia treatment. Trial methods were appropriate and accepted by families.
